# Outbreak of *Mycoplasma pneumoniae* at a military academy

**DOI:** 10.1186/s40779-020-00289-x

**Published:** 2020-12-03

**Authors:** Xin Zhang, Min-Na Han, Jing-Hui Dong, Xiao-Xi Li, Xian-Yun Hu, Zhi Wang, En-Qiang Qin, Jing Li, Jun-Yuan Tan, Fu-Sheng Wang, Lei Huang

**Affiliations:** 1 Center for Infectious Disease, the Fifth Medical Center of Chinese PLA General Hospital, Beijing, 100039 China; 2 Southern Distract Health Service of Chinese PLA General Hospital, Beijing, 100039 China; 3 Radiology Department, the Fifth Medical Center of Chinese PLA General Hospital, Beijing, 100039 China; 4 Clinical Lab Center, the Fifth Medical Center of Chinese PLA General Hospital, Beijing, 100039 China; 5 Medical Service Department, the Fifth Medical Center of Chinese PLA General Hospital, Beijing, 100039 China

**Keywords:** *Mycoplasma pneumoniae*, Epidemiological survey, Disease control and prevention

## Abstract

In 2019, an outbreak of *Mycoplasma pneumoniae* occurred at a military academy in China. The attack rate (10.08%,60/595) was significantly different among the units. High-intensity training and crowded environments to which cadets are exposed are the high risk factors for the outbreak of *M. pneumoniae*. In-time prevention and control measures effectively controlled the spread of the epidemic.

**Supplementary Information:**

The online version contains supplementary material available at 10.1186/s40779-020-00289-x.

Dear editor,

*Mycoplasma pneumoniae* may be the causative agent of 70% in crowded populations such as military personnel and students [1]. In the winter of 2019, a *M. pneumoniae* outbreak occurred at a military academy in China. Here we report the result of epidemiological investigation and disease prevention/control measures.

The outbreak occurred among training freshmen, the barracks were devoid of heating equipment, with residence area of 2.8–3.2 m^2^ per capita. All cadets ate at one cafeteria. Since late November 2019, total 15 cadets developed cold-like symptoms, all of them tested positive for *M. pneumoniae* IgM antibody, and several had CT scan-confirmed pneumonia. As the outbreak continued to spread, the patients were isolated in a temporary ward in early December. As strict prevention and control measures were timely implemented, new cases quickly declined, and the last case was reported on January 1st 2020.

The attack rate was 10.08% (60/595), with a total of 36 confirmed cases and 24 suspected cases (case definition see Additional file: Appendix I). The outbreak lasted 102 days, with four peaks. The interval between each peak was nearly the incubation period (Fig. 1).

**Fig. 1:**
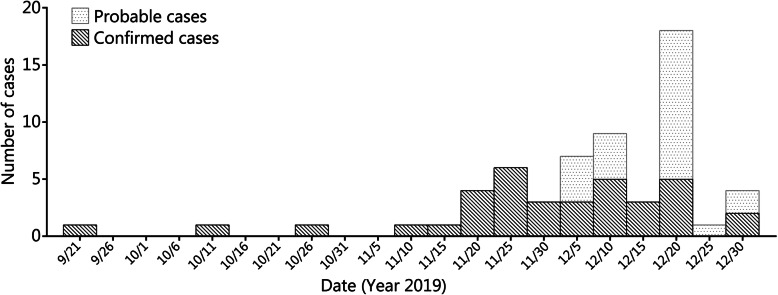
Number of confirmed cases every 5 days

The 60 cases were distributed among five companies:12 in company G, 10 in company H, 17 in company I, four in company J, and 17 in company K. The highest attack rate was in company K (17/110, 15.45%) and the lowest in company J (4/118, 3.39%). There were significant differences in attack rate between companies H and K (χ^2^ = 3.515, *p* = 0.048), G and J (χ^2^ = 3.871, *p* = 0.042), I and J (χ^2^ = 9.351, *p* = 0.002), and J and K (χ^2^ = 9.910, *p* = 0.001; Additional file: Appendix Table S1 and Appendix Figure S1).

We obtained complete medical records for 33 cases, including 19 confirmed and 14 suspected cases. The most common symptoms were cough (30/33) and fever (27/33). Other symptoms included runny nose (27, 81.82%), rhinobyon (23, 69.70%), sore throat (22, 66.67%), sneezing (21, 63.64%), fatigue (21, 63.64%) and headache (18, 54.55%). Sixteen cases had a history of burnout (e.g., stay up late or night shifts).

Within the 36 cases that tested positive for IgM antibody. Thirty-three of the patients had a chest computed tomography (CT) scan and 21 of the patients had radiologically confirmed pneumonia. The main damages were consolidation in 8 cases, ground glass opacity in 8 cases and fibrosis formation in 2 cases. Details see Additional file: Appendix Figure S1.

All confirmed and suspected cases were treated with moxifloxacin or levofloxacin for 10 to 14 days. Two severe cases were hospitalized for 7 to 10 days. All patients were successfully treated without any complications or sequela.

Following the incidence of febrile patients, an emergency response team was immediately organized. The prevention and control measures were the following. (1) Isolation: all patients were isolated in a temporary ward, and all group activities were canceled. (2) Quarantine: all close contacts were quarantined. (3) Daily morning health check for any symptoms of disease. (4) Daily “zero” reporting system. (5) Sterilization and disinfection, especially in dormitories, bathrooms, and cafeteria. (6) Health education: wear face masks, improve hand hygiene and cough manners (cover mouth and nose with elbow or tissue instead of hands). Following a maximum incubation period with no registered new cases, the outbreak was declared terminated.

Clinical manifestations of *M. pneumoniae* infections are variable. The most common manifestation is tracheobronchitis. Most of patients report non-specific symptoms similar to those of the common cold or other upper respiratory infections (URI). It is a challenge to effectively distinguish *M. pneumoniae* pneumonia from pneumonia caused by other pathogens based on clinical manifestations or imaging results [2]. Currently, the most common clinical tests are based on serology and nucleic acid detection. The titer of IgM antibody reaches a detectable level within 1 week after disease onset, and the sensitivity is low, limiting the clinical value of these tests in early diagnosis [3].

Currently, macrolides are the preferred first-line antibiotics for *M. pneumoniae* infections. However, surveillance of antibiotic resistance by Chinese CDC showed that the prevalence of macrolide-resistant *M. pneumoniae* (MRMP) was 98.1% in children under 14 years of age and 83% in adolescents and adults [4]. Respiratory fluoroquinolones can achieve high concentrations in lung tissue and have greater activity than macrolides.

This outbreak in Northern China lasted more than 3 months. The time interval between each peak, which was approximately an incubation period, showed the typical features of generation to generation transmission. The possible reasons for this outbreak may be explained as follows, 1) high-intensity training and psychological stress decreases immunity and 2) cold and dry climates, high-density residences, and non-ventilated rooms promote the epidemic spread.

In summary, accurate etiological diagnosis, early isolation, standardized treatment of patients, medical observation, quarantine of close contacts, and regular environmental disinfection are crucial measures to reduce secondary transmission and mitigate outbreak consequences.

## Supplementary Information

**Additional file**.

## Data Availability

All data generated or analyzed during this study are included in this published manuscript.

## Abbreviations

CAP: Community-acquired pneumonia
CT: Computed tomography
URI: Upper respiratory infections
MRMP: Macrolide-resistant *M. pneumoniae*
