# Supplementary material for: Outbreak of Mycoplasma pneumoniae at a military academy
Source: Mil Med Res. 2020 Dec 3;7:60. doi: 10.1186/s40779-020-00289-x (PMC7713002; doi:10.1186/s40779-020-00289-x)
Supplement: Supplementary file 1 — Additional file. [file 40779_2020_289_MOESM1_ESM.docx]

**Additional file 1** Case definition

We developed a case definition based on history of contact, clinical manifestations, and available laboratory tests as follows: (1) Suspected case: history of contact with a confirmed case within the past three weeks; presenting cold-like symptoms including sore throat, dry or productive cough, headache, coryza, and fever. (2) Confirmed case: presenting cold-like symptoms including sore throat, dry or productive cough, headache, coryza, and fever; testing positive for M. pneumoniae IgM antibody. (3) Close contact: shared a dormitory; came into close proximity (i.e., within ~1.5 m) of a confirmed case or had direct/indirect contact with the secretion of a confirmed case within the past three weeks.

**Additional file Table S1** Distribution of cases within the military campus

| Unit | Number of cadets | Number of cases | Attack rate (%） |
| --- | --- | --- | --- |
| Company G | 124 | 12 | 9.68 |
| Company H | 129 | 10 | 7.75 |
| Company I | 114 | 17 | 14.91 |
| Company J | 118 | 4 | 3.39 |
| Company K | 110 | 17 | 15.45 |
| Total | 595 | 60 | 10.08 |

**Additional file Figure S1** Location of the cadets’ dormitory and other facilities.

**

**

A: Learning and teaching building; B: Office of cadet brigade; C: Cafeteria; D: Infirmary; G: Company G; H: Company H; I: Company I; J: Company J; K: Company K; L: Company L (no case).

**Additional file Figure S2** Lung CT images of four cases infected with *Mycoplasma pneumoniae.*





White arrows in (a): Ground glass opacity; in (b): Increased density; in (c): Consolidation; in (d): Fibrosis formation.
